# Supplementary material for: Estimating cortical thickness trajectories in children across different scanners using transfer learning from normative models
Source: Hum Brain Mapp. 2024 Feb 5;45(2):e26565. doi: 10.1002/hbm.26565 (PMC10839740; doi:10.1002/hbm.26565)

Supplementary Figure 1: In total 2238 scans were excluded, most of them due to low quality ratings. Here we list reasons for exclusions in detail.


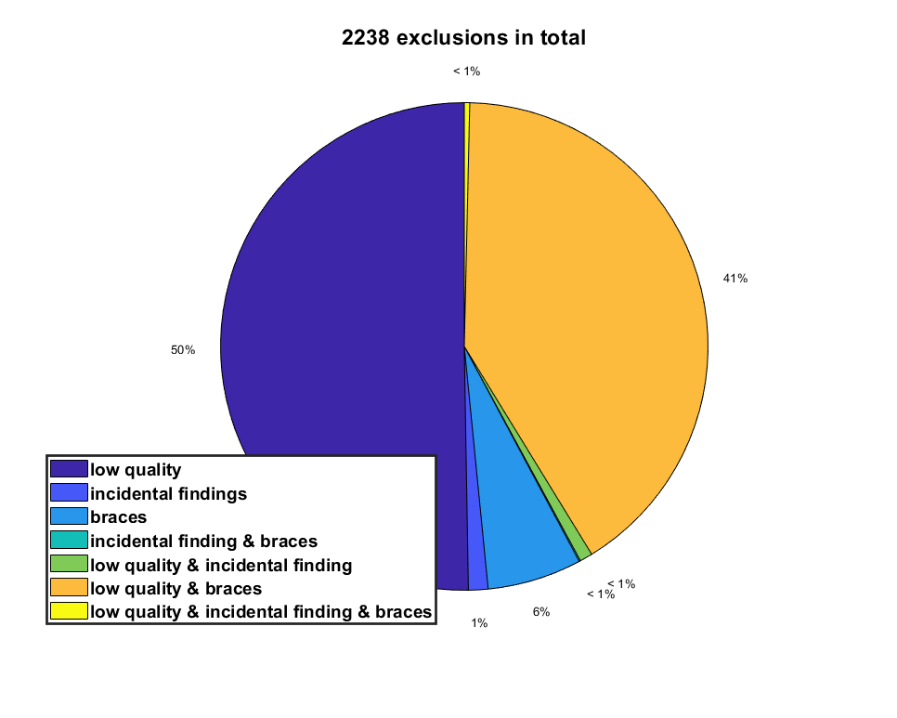


Supplementary Figure 2: We calculated Pearson correlations between model metrics and ROI area (as reported by Destrieux et al., 2010). We find small but significant correlations with larger ROIs outperforming smaller ROIs. Correlation are similar when adaptation sets contained A) 25 scans, and B) 100 scans.


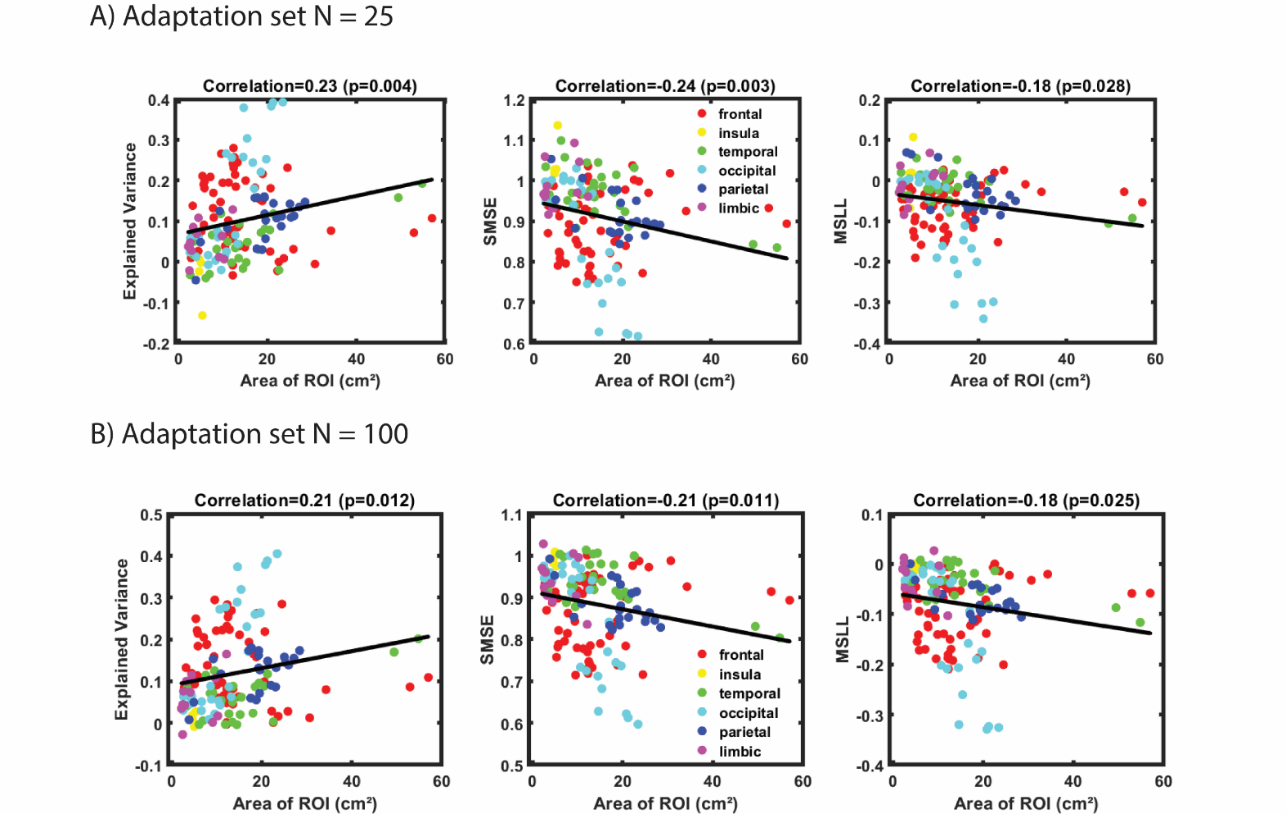


Supplementary Figure 3.: Effects of different recalibration configurations on the target cohort illustrated in all ROIs of the Destrieux parcellation. ROIs are grouped in frontal, parietal, temporal, insular and limbic, and occipital lobes.


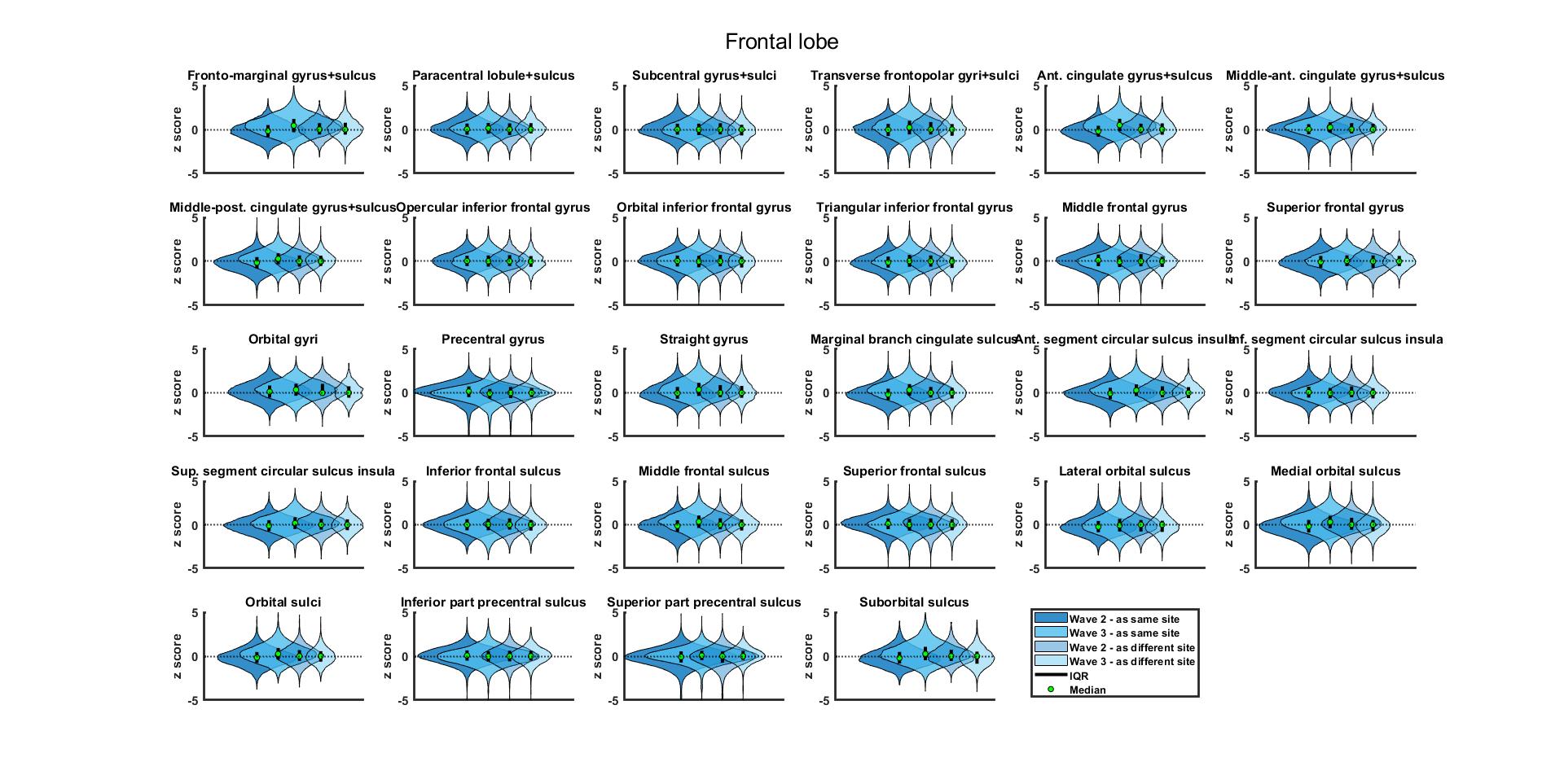


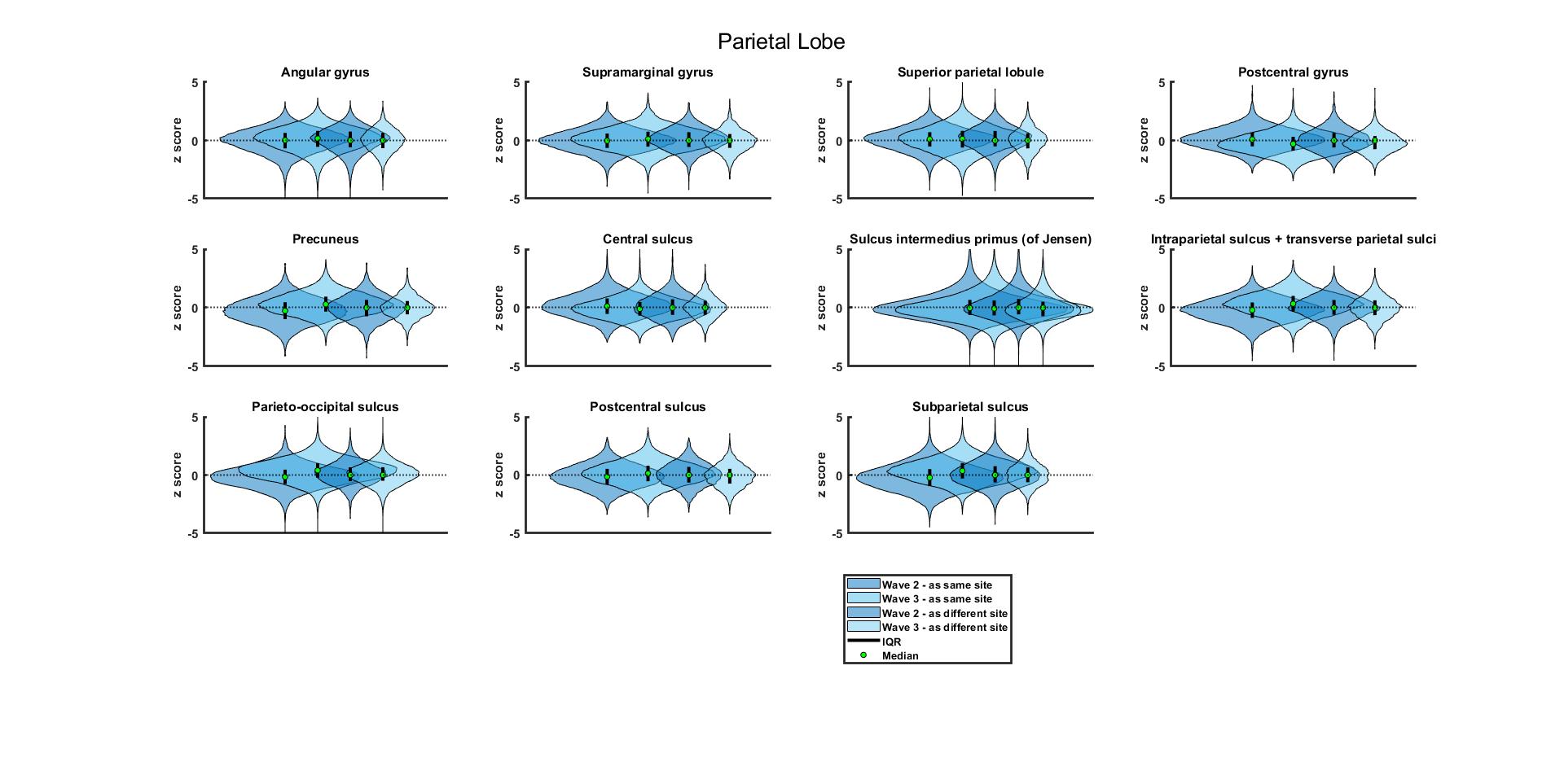


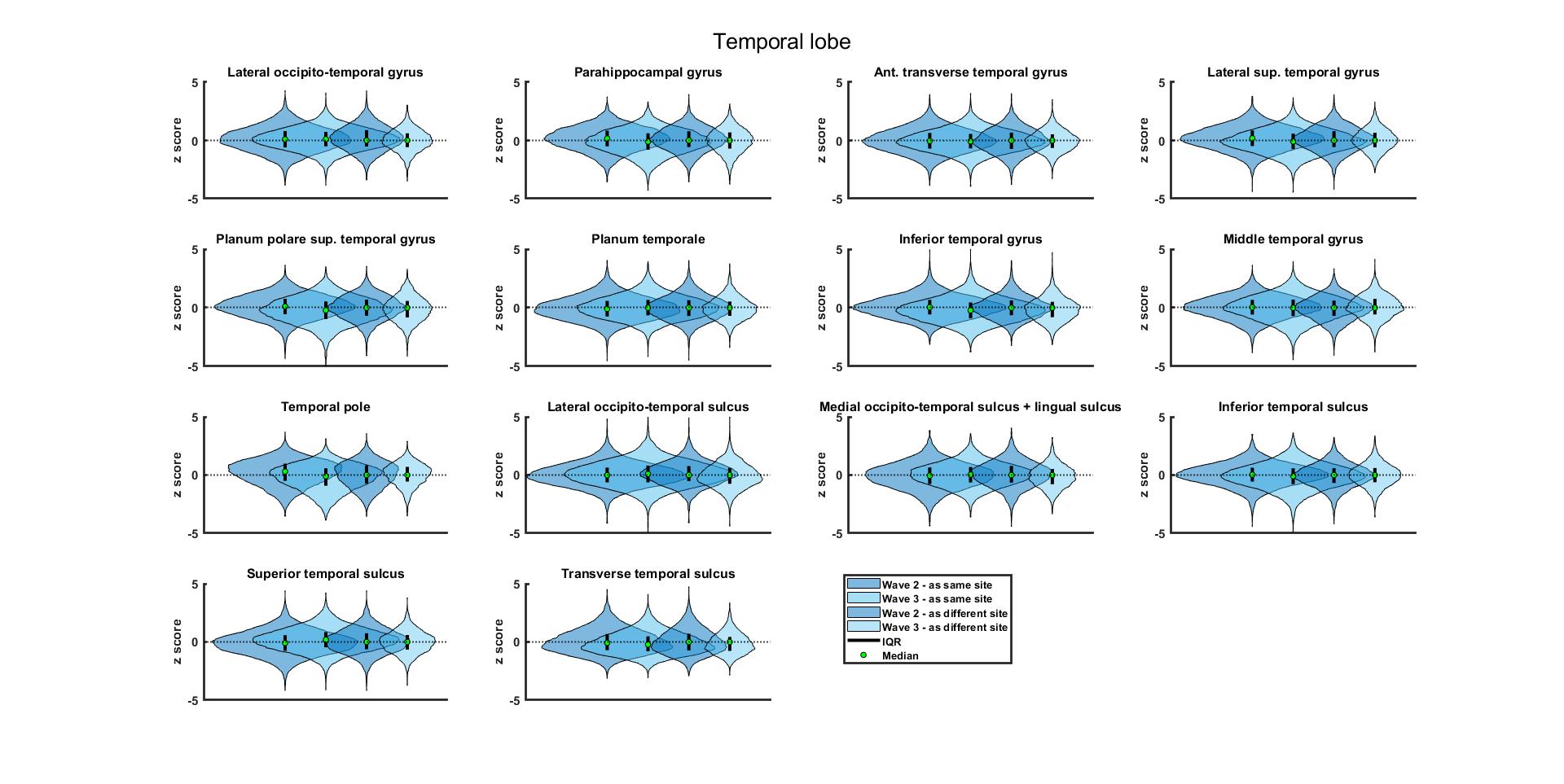


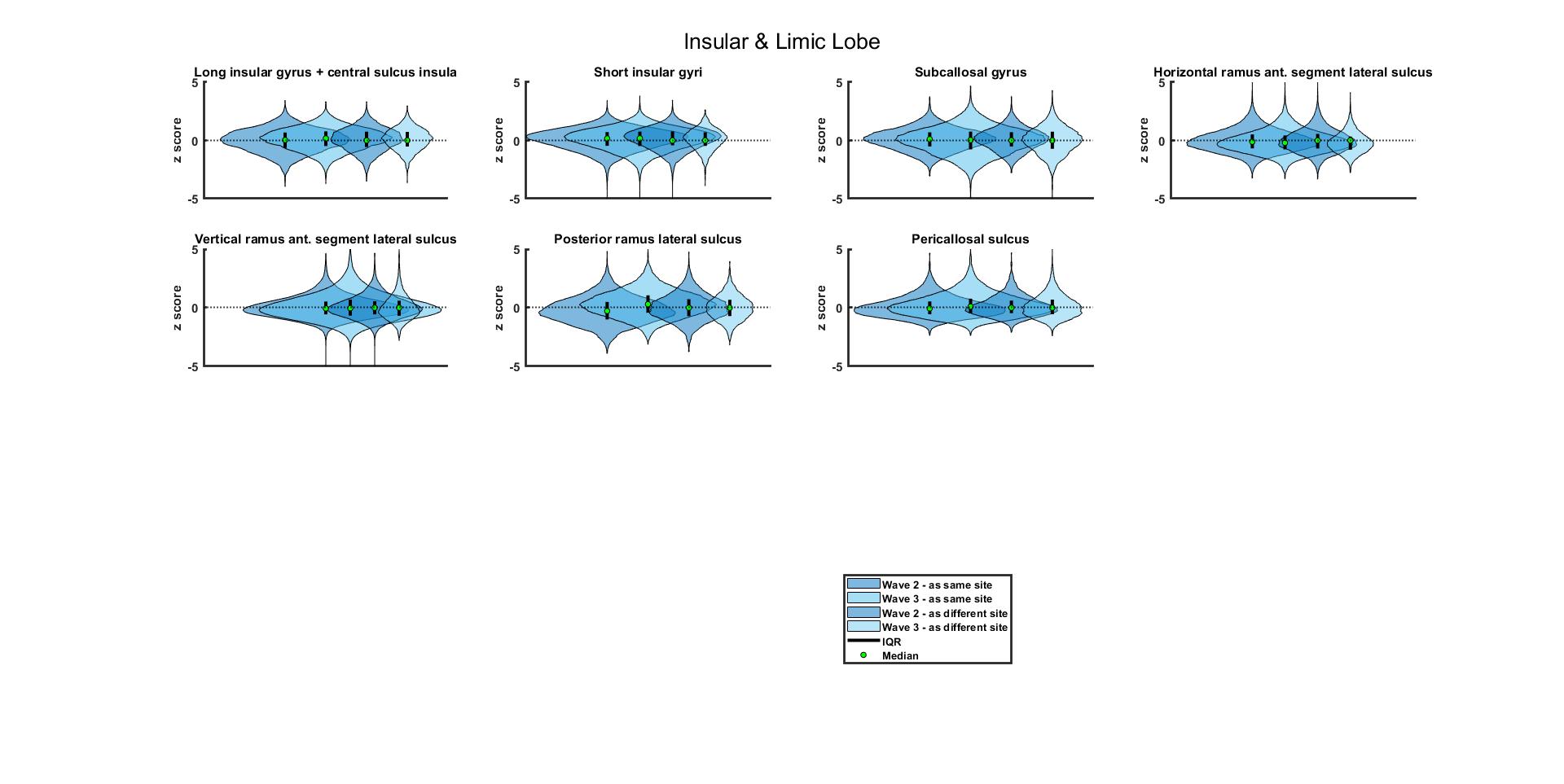

Supplement: Supplementary file 1 — SUPPLEMENTARY FIGURE 1: In total 2238 scans were excluded, most of them due to low quality ratings. Here we list reasons for exclusions in detail. SUPPLEMENTARY FIGURE 2: We calculated Pearson correlations between model metrics and ROI area (as reported by Destrieux et al., 2010). We find small but significant correlations with larger ROIs outperforming smaller ROIs. Correlation are similar when adaptation sets contained (A) 25 scans, and (B) 100 scans. SUPPLEMENTARY FIGURE 3: Effects of different recalibration configurations on the target cohort illustrated in all ROIs of the Destrieux parcellation. ROIs are grouped in frontal, parietal, temporal, insular and limbic, and occipital lobes. [file HBM-45-e26565-s001.docx]
